# Polarization-controlled optimal scatter suppression in transient absorption spectroscopy

**DOI:** 10.1038/srep43484

**Published:** 2017-03-06

**Authors:** Pavel Malý, Janneke Ravensbergen, John T. M. Kennis, Rienk van Grondelle, Roberta Croce, Tomáš Mančal, Bart van Oort

**Affiliations:** 1 Biophysics of Photosynthesis, Department of Physics and Astronomy, Faculty of Sciences, and LaserLaB Amsterdam, Vrije Universiteit Amsterdam, 1081 HV, Amsterdam, The Netherlands; 2 Institute of Physics, Faculty of Mathematics and Physics, Charles University, Czech Republic

## Abstract

Ultrafast transient absorption spectroscopy is a powerful technique to study fast photo-induced processes, such as electron, proton and energy transfer, isomerization and molecular dynamics, in a diverse range of samples, including solid state materials and proteins. Many such experiments suffer from signal distortion by scattered excitation light, in particular close to the excitation (pump) frequency. Scattered light can be effectively suppressed by a polarizer oriented perpendicular to the excitation polarization and positioned behind the sample in the optical path of the probe beam. However, this introduces anisotropic polarization contributions into the recorded signal. We present an approach based on setting specific polarizations of the pump and probe pulses, combined with a polarizer behind the sample. Together, this controls the signal-to-scatter ratio (SSR), while maintaining isotropic signal. We present SSR for the full range of polarizations and analytically derive the optimal configuration at angles of 40.5° between probe and pump and of 66.9° between polarizer and pump polarizations. This improves SSR by 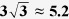 (or 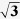 compared to polarizer parallel to probe). The calculations are validated by transient absorption experiments on the common fluorescent dye Rhodamine B. This approach provides a simple method to considerably improve the SSR in transient absorption spectroscopy.

Optical spectroscopy has been indispensable in the progress of a plethora of scientific fields. One particularly powerful implementation is transient absorption spectroscopy (TA), in which a first pulse (“pump”) induces a photoprocess, and a second (“probe”) monitors the changes of the sample’s optical properties^1^,^2^,^3^. TA is used to monitor diverse photoinduced processes, such as electron and energy^4^,^5^,^6^,^7^,^8^,^9^,^10^,^11^,^12^,^13^,^14^ transfer, proton transfer^15^,^16^, isomerizations^17^,^18^, addition reactions^2^, and DNA repair^19^,^20^.

Scattered pump (excitation)-light can strongly distort signals in ultrafast TA (and in many other types of spectroscopy), in particular when detecting close to the pump frequency. Several approaches are used to reject scatter, such as optical shielding, increasing optical path length between sample and detector, and rejecting the probe signal around the pump frequency^1^,^3^,^12^,^21^. A common approach involves the use of polarized pump and probe pulses, combined with a polarizer in the probe beam behind the sample. In this realization the polarization between pump and probe pulses is usually set at magic angle (MA, ~54.7°), to ensure isotropic signals. Introducing a polarizer set perpendicular to the pump polarization (i.e. at 54.7°–90° to the probe) will almost completely suppress the (polarized) pump scatter. Importantly, however, the polarizer will re-introduce anisotropy in the probe signal^22^ (see also below). Thus, scatter rejection comes at the cost of loss of signal isotropy, introducing anisotropic spectrokinetics. Introducing a polarizer set parallel to the probe (i.e. at MA to the pump) maintains both the signal amplitude and isotropy and reduces the scatter by 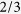. This indicates an interesting possible route to decrease the unwanted scatter even more, by changing the combination of the probe polarization and polarizer direction.

We therefore set out to find conditions to combine the benefits of these two approaches, and to use the degree of freedom that was ignored so far: the polarization angle between pump and probe. The idea behind this approach is that at any angle other than MA between pump and probe polarization the signal is anisotropic, but that the isotropic signal can be retrieved by a polarizer set at the correct polarization. Different pump polarization/probe polarizer settings yield different signal and scatter amplitudes (taking advantage of the fact that the scattered pump light is predominantly polarized as the pump). We analytically determine the setting that optimizes both the signal and the signal-to-scatter ratio (SSR). The scatter is reduced by 85%, whereas only 20% of signal amplitude is lost, thus improving SSR by a factor of 5.2 as compared to measuring without a polarizer (and by a factor of 1.7 compared to a polarizer parallel to the probe). We quantitatively confirm our numerical analysis by fs-transient absorption spectroscopy on Rhodamine B, a frequently used fluorescent dye.

Thus, we provide a simple method to control the signal-to-scatter ratio in ultrafast transient absorption spectroscopy. An analogous approach is generally applicable in other four-wave mixing experiments. Thus, we provide a method to reduce scatter in e.g. time-resolved stimulated Raman^23^, pump-repump/dump-probe^12^ and 2D electronic spectroscopy^24^ experiments.

## Results

### Theoretical considerations

Transient absorption measurement is a nonlinear spectroscopic technique which probes the 3rd order macroscopic polarization *P*_*i*_(*ω*) resulting from the interaction of the sample with the pump ***E***_*P*_ and probe ***E***_*PR*_ fields:


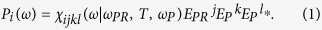


Here the *χ*_*ijkl*_(*ω* | *ω*_*PR*_,*T,ω*_*p*_) is the third-order susceptibility, which is a function of the delay *T* between the pump and probe and their frequencies *ω*_*P*_ and *ω*_*PR*_. The macroscopic polarization is connected to the signal through the Maxwell equations^25^,


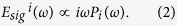


Because of phase matching, this signal field is generated in the *k*_*S*_ = *k*_*PR*_ + *k*_*P*_ − *k*_*P*_ = *k*_*PR*_ probe direction. In a typically considered experimental scheme, the signal and probe field are dispersed on a spectrograph and their intensity, i.e. the square of the sum of their amplitudes, is detected^26^:


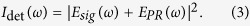


Subtracting the probe itself and neglecting the weak intensity of the signal alone, the measured transient absorption signal is proportional to


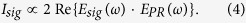


That is, the detection is self-heterodyned and the *projection* of the signal field onto the probe polarization is measured. The measured part of the sample response is


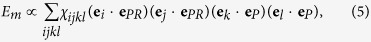


where we dropped the frequencies in the response and denote unity polarization vectors **e**_*PR*_, **e**_*P*_ and unity basis vectors **e**_*i*_. In this case we have two projections on the pump and two on the probe field. Starting from this expression the usual isotropic magic angle and anisotropy measurement conditions can be derived, even for general pump and probe polarizations^27^.

However, for experimental reasons, mostly removal of unwanted signals such as scattering from the pump, it is often useful to insert a polarizer *after* the measured sample. In this case, both the signal and probe field polarizations are projected on the plane of the polarizer **e**_*PO*_. For the probe field it means just a decrease in amplitude: *E*_*PR*_**e**_*PR*_ → *E*_*PR*_(**e**_*PR*_ · **e**_*PO*_)**e**_*PO*_. But for the signal field this changes the contribution of the elements of the response, which yields the measured signal


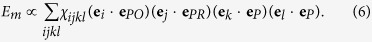


This form of a measured signal field leads to different conditions on the polarizations of the fields for isotropic magic angle or anisotropy measurement. Let us now derive these conditions for an isotropic sample of randomly-oriented particles. In that case the macroscopic response is given by averaging the response of the individual particles over all their possible orientations. After such orientational averaging, it turns out that only 21 elements of the macroscopic response are non-vanishing: χ_*iiii*_, χ_*iijj*_, χ_*ijij*_, χ_*ijji*_ (ref. 27 and ref. therein), out of which only three are independent. Let us consider a case of nearly-parallel-propagating pump and probe beams (for practical reasons such as interaction length this is often the case, for more general treatment see e.g. ref. 27). Without loss of generality we can choose the beam propagation direction in the laboratory frame in *z* – direction. The pump will be linearly polarized in the *x* – direction, **e**_*P*_ = **e**_*x*_. The (projected) signal field is then


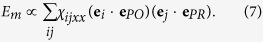


Because of the orientational averaging and the polarization vectors lying in the *xy* – plane, only two components of the response have to be considered χ_*xxxx*_ and χ_*yyxx*_. These can be expressed by the individual particle response *κ*_*abcd*_^27^:


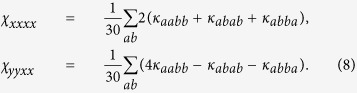


The first term in the brackets, *κ*_*aabb*_, represents the isotropic contribution while the second two terms include the anisotropy. Writing Eq. (7) in components, we have


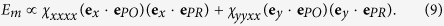


Evidently the *x* – and *y* – projection of the probe and polarizer determine the weights with which the two parts of the response contribute to the signal. By choosing these properly, we can, for instance, eliminate the anisotropy part by demanding that


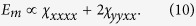


Let us use this as a condition for an isotropic signal:


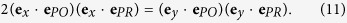


If we measure the probe polarization angle *ϕ*_*PR*_ and polarizer angle *ϕ*_*PO*_ relative to the pump polarization, we can express this condition as


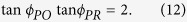


Note that this equation is also given as an ‘academic exercise’ in ref. 22. Solving Eq. (11) for the probe polarization dependent on the polarizer direction, we get


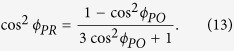


For ϕPR = ϕPO this gives the magic angle condition For any desired polarizer direction we can now set the probe polarization to obtain only the isotropic part of the signal.

Unfortunately, as we will see below, some particularly interesting polarizer directions, such as perpendicular to the pump (which would completely eliminate the scatter), do not work, because they yield zero signal. We can, however, adjust the polarizer plane to obtain the optimal signal and signal-to-scatter ratio (SSR).

In many cases pump scattering is a major source of noise, and is preferably removed. Let us therefore calculate the SSR. The scatter intensity is proportional to the portion of the pump which passes the polarizer:


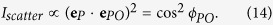


Because the measured pump-probe signal is obtained by mixing the signal and probe fields, Eq. (4), the signal intensity is given by the signal and probe fields after the polarizer. By applying the isotropic condition Eq. (11), to Eq. (9) we can see that the signal is proportional to 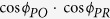. The transient absorption signal intensity is then


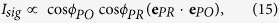


where the term in the brackets is the projection of the probe field on the plane of the polarizer. The geometrical dependence of the signal to scatter ratio (SSR) is thus given by


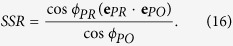


**Figure 1:**
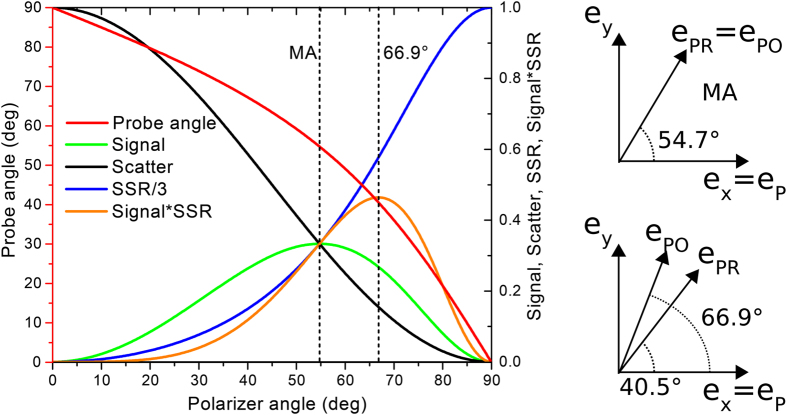
Left: Dependence of isotropic angle conditions on the angle of the polarizer after the sample. Red (left axis): corresponding probe angle to obtain isotropic signal. Further is depicted (right axis) the signal amplitude, (Eq. (15), green), scatter amplitude, (Eq. (14), black), signal to scatter amplitude, (Eq. (16), blue). The desired optimized quantity of signal multiplied by SSR is depicted in orange. Two particular polarizer angles used in this work, traditional magic angle 54.7° and the optimal scatter suppression angle 66.9° are denoted by vertical dashed lines. In absence of a polarizer, with probe angle 54.7°, the values are: Signal = 1/3, Scatter = 1, SSR = 1/3, Signal*SSR = 1/9. **Right**: Orientation of the polarization vectors for the case of traditional magic angle 54.7° (top) and the optimal scatter suppression angles (bottom).

The signal, scatter, SSR and probe angle yielding isotropic signal are depicted in Fig. 1 for different polarizer angles (relative to the pump polarization). For the polarizer perpendicular to the pump, we get complete noise suppression but no isotropic signal. For the opposite case of polarizer parallel with the pump, we get only scatter. The signal has a maximum for the polarizer oriented in parallel with the probe, where the probe is at the usual magic angle 54.7°. In this setting no signal intensity is lost compared to the measurement without the polarizer, while the noise is reduced by 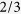, increasing SSR 3-fold. This is already a significant improvement, advocating the use of the polarizer. However, there is room for further improvement. Ideally one wants to have *both* high SSR and high signal amplitude. We thus plot also the product *SSR*⋅*signal*. This function has a maximum at 66.9°, which is an optimal angle for the polarizer to have highest signal and SSR. The corresponding probe polarization angle to the pump is 40.5°. In this setting SSR is 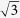 times larger than with the polarizer parallel to the probe at the conventional magic angle and thus 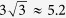 times larger than when measuring without the polarizer. In the case of a strong signal the polarizer angle can be further increased, yielding even higher SSR. This is, however, at the expense of the signal intensity.

### Transient absorption of Rhodamine B

**Figure 2:**
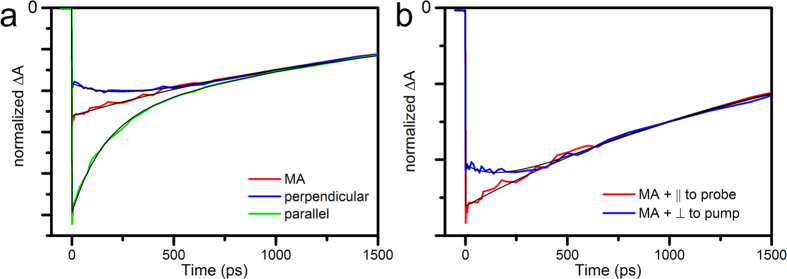
Transient absorption spectroscopy of Rhodamine B, with varying polarization angle between pump and probe. Kinetic traces probed at 568 nm (normalized at 3 ns). (**a**) Polarizer behind the sample set parallel to the probe polarization. Polarization angle between pump and probe was set to magic angle (54.7°, red), perpendicular (blue) or parallel (green). (**b**) Polarizer behind the sample set parallel to the probe (red) or perpendicular (blue) to the pump polarization. Polarization angle between pump and probe set to magic angle. Black lines: exponential fits of the data. Fitted rates in (**a**) were 1.8 ns (magic angle), 0.2 ns and 1.8 ns (perpendicular), 0.2 ns and 1.7 ns (parallel), and in (**b**) 1.8 ns (parallel to probe), 0.2 ns and 1.8 ns (perpendicular).

We start with a careful check of the effect of a polarizer behind the sample on the signal (an)isotropy in transient absorption spectroscopy. We used the common fluorescent dye Rhodamine B in water, with femtosecond 520 nm excitation (pump) pulses and broadband visible probe pulses. Figure 2a shows the kinetics of the ground state bleach at 568 nm. For magic angle conditions the data can be fitted with a single exponential decay of 1.8 ns, assigned to the decay of singlet excited Rhodamine B^28^. For parallel or perpendicular polarization angle of pump and probe an extra rate constant of 0.2 ns is found. This fast rate is assigned to the loss (parallel) or growth (perpendicular) of signal due to anisotropy decay caused by molecular rotation^28^.

Figure 2b shows that for the magic angle case similar 0.2 ns kinetics are introduced when a polarizer after the sample is set perpendicular to the pump polarization. This data illustrates that this polarization scheme is not suited for anisotropy free measurements. Note that the detection optics after the sample can also be polarization sensitive, and consequently introduce anisotropy signals in the same way. However, with a polarizer after the sample at the correct angle, any polarization sensitive detections optics will only reduce signal amplitude, but not introduce anisotropy. This is one reason to include a polarizer after the sample even when measuring under the traditional magic angle. The polarizer is then parallel with the probe polarization.

**Figure 3:**
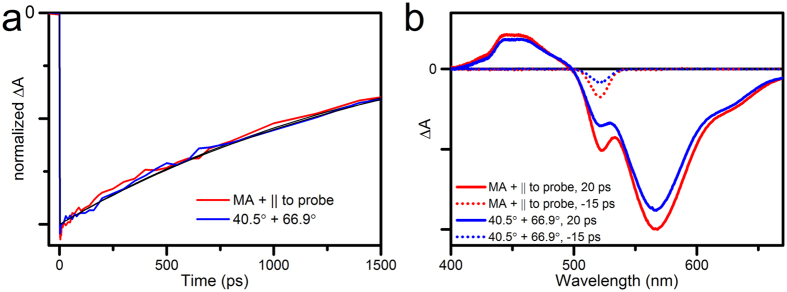
Transient absorption spectroscopy of Rhodamine B, with two combinations of pump-probe angle and polarizer angle. (**a**) Kinetic traces probed at 568 nm (normalized to the fitted value at t_0_). Pump-probe polarization was at magic angle and polarizer parallel to probe (red) or pump-probe polarization at 40.5°, polarizer at 66.9° with respect to pump (blue). Black lines: exponential fits of the data with rates of 1.6 ns (MA + parallel) and 1.7 ns (40.5° + 66.9°). (**b**) Time-gated difference spectra (raw data, measured at equal pump power) at t = 10 ps and at t = −15 ps (dotted lines); colour coding as in (**a**). Full datasets are shown in Fig. 4.

In the theoretical section we proposed polarization angles of 40.5° between pump and probe, and 66.9° between pump and polarizer for optimal scatter suppression. This combination indeed yields the single exponential, anisotropy-free decay kinetics of Rhodamine B (Fig. 3a).

**Figure 4:**
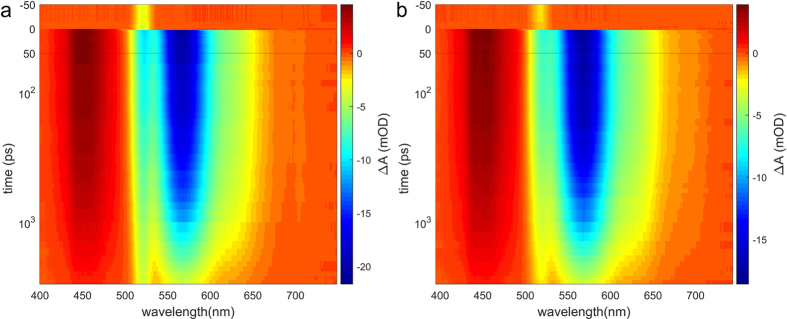
Transient absorption spectroscopy of Rhodamine B, with two combinations of pump-probe angle and polarizer angle. The continuous signal around 520 nm is caused by pump scatter. (**a**) pump-probe polarization at magic angle, polarizer parallel to probe. (**b**) pump-probe polarization at 40.5°, polarizer at 66.9° with respect to pump. The time axis is linear from −50 to 50 ps and logarithmic from 50 ps to 3 ns. The colour scales are adjusted to the amplitude of the transient absorption signal. The relative amplitude of the scatter can be estimated at negative delays.

Now that it is clear that the polarizer recovers the isotropic signal, we study its effect on the signal-to-scatter ratio (SSR), which we theoretically predicted to increase compared to the case with magic angle and polarizer parallel to probe. This prediction is confirmed by the experimental data. The relative amplitude of scatter is most obvious at negative delays, where there is no overlap with transient absorption signal. Indeed, time-gated spectra before t_0_ show a clear decrease of the scatter amplitude of the 40.5°/66.9° combination compared to MA/parallel (Fig. 3b dotted lines). In other words, pump-scatter is effectively suppressed. This suppression is also visible at 20 ps, where the scatter appears as a peak on top of the transient signal (Fig. 3b, continuous lines). As predicted, the transient signal is slightly weaker than under MA conditions. These observations are confirmed in the full datasets (Fig. 4, colour scales adjusted to signal amplitude).

To quantify the signal to scatter ratio, the amplitude of the scatter is measured at the pump wavelength and the amplitude of the signal at the maximum bleach wavelength. For scatter amplitudes, the data at negative delays is used, where the scatter is not overlapped by transient absorption signal. The amplitude at all negative delays is averaged. This gives a scatter amplitude of 3.5 +/− 0.1 mOD for magic angle and 1.7 +/− 0.1 mOD for the combination of 40.5° and 66.9°. For signal amplitudes, all positive delays are averaged (weight-corrected for the exponential decay). This gives signal amplitudes of 20.4 +/− 0.9 mOD and 17.9 +/− 0.4 mOD respectively. From this it follows that the improvement of the signal to scatter ratio is 1.8 +/− 0.1, in agreement with the predicted value of 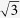.

## Conclusion

By employing a polarizer after the sample in transient absorption spectroscopy, adverse scattered excitation light can be considerably suppressed. At the same time, such a polarizer can introduce anisotropy in the measured signal. We have theoretically derived all combinations of probe polarization angles and polarizer orientations for which an isotropic signal is obtained. We show, theoretically and experimentally, how the signal-to-scatter ratio depends on the polarizer, and we find an optimal angle for scatter suppression. This simple polarization configuration improves the signal-to-scatter ratio 5.2-fold. At the same time, the polarizer prevents anisotropy effects introduced by the polarization sensitive detection optics.

With this work, we emphasize the important role of the polarizer after the sample. It demonstrates the extreme utility of such a polarizer in scatter suppression, showing how to make maximum use of it. At the same time, it serves as a word of caution, as careless polarizer placement can introduce anisotropy in the measurement, with the risk of data misinterpretation.

## Methods

### Transient absorption spectroscopy.

TA was performed on a previously described setup^11^, using a Ti:sapphire laser amplifier (Libra, Coherent, Santa Clara, CA). The amplifier was seeded by an 80 MHz oscillator (Vitesse, Coherent) and pumped with an Nd:YLF laser (Evolution, Coherent). The amplifier generates 800 nm pulses with a duration of ~40 fs and a repetition rate of 1 kHz, which drive an optical parametric amplifier (OperA SOLO, TOPAS, Coherent), generating 520 nm excitation (pump) pulses. The excitation energy was set to 50 nJ per pulse, focused to a spot size of 140 μm. A broadband probe beam was generated by focusing part of the output of the laser amplifier on a CaF_2_ plate.

The pump polarization was set by a Berek polarization compensator followed by a polarizer. A second polarizer was placed in the probe path before the sample and set parallel to original probe polarization, to ensure a clean polarization of the probe. The pump and probe were overlapped on the sample in a near-collinear configuration. After the sample the pump is blocked and the probe and transient absorption signal are dispersed by a prism and recorded on a CCD detector (Entwicklungsbüro Stresing, Berlin, Germany). A third polarizer was placed in the probe path between the sample and detector. Transient absorption data was recorded with delays up to 3 ns. The data were fitted by global analysis using the program Glotaran (v1.5.1). Rhodamine B (Sigma) was solubilized in deionized water (Milli-Q, Millipore Corporation), in a shaking 1 mm path length quartz cuvette, at an OD of 0.45 at the peak absorption wavelength.

## Additional Information

**How to cite this article:** Malý, P. *et al*. Polarization-controlled optimal scatter suppression in transient absorption spectroscopy. *Sci. Rep.* **7**, 43484; doi: 10.1038/srep43484 (2017).

**Publisher's note:** Springer Nature remains neutral with regard to jurisdictional claims in published maps and institutional affiliations.
